# Stability Despite Reduction: Flower Structure, Patterns of Receptacle Elongation and Organ Fusion in *Eriocaulon* (Eriocaulaceae: Poales)

**DOI:** 10.3390/plants9111424

**Published:** 2020-10-24

**Authors:** Dmitry D. Sokoloff, Shrirang R. Yadav, Arun N. Chandore, Margarita V. Remizowa

**Affiliations:** 1 Biological Faculty, M.V. Lomonosov Moscow State University, 119234 Moscow, Russia; dmitry.sokoloff@msu-botany.ru or; 2 Shivaji University, Vidyanagar, Kolhapur 416 004, MS, India; sryadavdu@rediffmail.com; 3 Department of Botany, Abasaheb Marathe Arts and New Commerce, Science College, Rajapur 416 702, District Ratnagiri, MS, India; arunchandore@gmail.com

**Keywords:** common primordia, Eriocaulaeae, *Eriocaulon redactum*, floral development, floral evolution, flower, monocots, organ fusion, receptacle, Poales

## Abstract

Eriocaulaceae (Poales) differ from potentially related Xyridaceae in pattern of floral organ arrangement relative to subtending bract (with median sepal adaxial). Some Eriocaulaceae possess reduced and non-trimerous perianth, but developmental data are insufficient. We conducted a SEM investigation of flower development in three species of *Eriocaulon* to understand whether organ number and arrangement are stable in *E. redactum*, a species with a highly reduced calyx and reportedly missing corolla of female flowers. Early flower development is similar in all three species. Male and female flowers are indistinguishable at early stages. Despite earlier reports, both floral types uniformly possess three congenitally united sepals and three petals in *E. redactum*. Petals and inner stamens develop from common primordia. We assume that scanning electron microscopy should be used in taxonomic accounts of *Eriocaulon* to assess organ number and arrangement. Two types of corolla reduction are found in Eriocaulaceae: suppression and complete loss of petals. Common petal–stamen primordia in *Eriocaulon* do not co-occur with delayed receptacle expansion as in other monocots but are associated with retarded petal growth. The ‘reverse’ flower orientation of *Eriocaulon* is probably due to strictly transversal lateral sepals. Gynoecium development indicates similarities of Eriocaulaceae with restiids and graminids rather than with Xyridaceae.

## 1. Introduction

**Figure 1:**
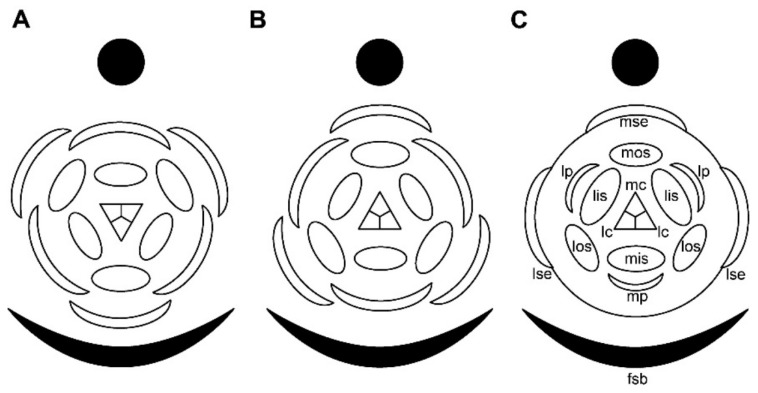
Flower diagrams. (**A**) Typical trimerous pentacyclic monocot flower with subtending bract and no floral prophylls. (**B**) Reverse orientation of trimerous pentacyclic flowers. (**A**,**B**) are generalized diagrams. (**C**) Flower diagram of *Eriocaulon* species studied here highlighting the fusion between sepals and pronouncedly transversal position of lateral sepals. Relative arrangement of all organs is the same in flowers of both sexes. This diagram can be used as a legend to abbreviations adopted in all subsequent figures. Closed circles indicate inflorescence axis, black arcs represent the flower-subtending bract, open arcs are perianth members, open ellipses are stamens, central triangle represents the gynoecium. The gynoecium is sterile in functionally male flowers and the stamens are sterile (staminodia) in functionally female flowers. fsb, flower-subtending bract; lc, lateral carpels; lis, lateral inner whorl stamens; los, lateral outer whorl stamens; lp, lateral petals; lse, lateral sepals; mc, median carpel; mis, median inner whorl stamen; mos, median outer whorl stamen; mp, median petal; mse, median sepal.

The occurrence of trimerous pentacyclic flowers (with 3 + 3 perianth members, 3 + 3 stamens and 3 carpels) is a stable feature of many monocot lineages and one of key characteristics of monocots as a natural group [1,2,3,4]. Orientation of trimerous pentacyclic flowers in relation to surrounding phyllomes shows stable patterns across monocots [1,5]. In particular, trimerous flowers of almost all monocots lacking floral prophylls (bracteoles) on lateral pedicels have a median outer whorl perianth member inserted abaxially and two other outer whorl perianth members in transverse-adaxial positions (Figure 1A). Exceptions from this rule are extremely rare and include the basal monocot family Araceae (Alismatales: [6]) and the commelinid family Eriocaulaceae (Poales: [1,7,8,9,10,11]), where the outer whorl median member of the trimerous perianth occupies an adaxial position (Figure 1B). However, Araceae are also remarkable in the total absence of flower-subtending bract (that plays important morphogenetic roles in other monocots) and overall high variation of flower groundplan [6,12]. In contrast, flower-subtending bracts are well developed at least in the peripheral flowers in Eriocaulaceae and trimerous pentacyclic flowers are common in one of the two largest genera of the family, *Eriocaulon* [9,13]. Therefore, trimerous flowers of Eriocaulaceae are nearly unique among monocots with respect to their orientation pattern.

Eriocaulaceae are well-known for their characteristic insect-pollinated compact racemose (partial) inflorescences. Surrounded by sterile involucral bracts, they resemble capitula of Asteraceae and some other specialized eudicots [11,14,15,16]. Similar compact inflorescences are found in another family of the order Poales, Xyridaceae, which may (or may not) be sister to Eriocaulaceae [17,18,19], but orientation of trimerous flowers follows the typical monocot pattern in Xyridaceae [11,20]. Although being tiny and uniformly colored, the perianth of Eriocaulaceae is believed to be differentiated into a calyx and a corolla. This feature is shared by Eriocaulaceae, Xyridaceae and another potentially related family, Mayacaceae [19,21,22], but in contrast with the two other families, members of Eriocaulaceae, with very few scattered reversals, possess functionally unisexual flowers [13,23,24].

The unusual nature of orientation of trimerous pentacyclic flowers in Eriocaulaceae highlights a need of more detailed comparative and developmental studies with attention to variation of flower groundplan in the family. The most obvious aspects of floral diversity in Eriocaulaceae are the reduction of the outer whorl of the androecium (its rudiments can be often traced in male flowers) in Paepalanthoideae, occurrence of dimery rather than trimery in some taxa, patterns of corolla tube formation and petal to stamen fusion [9,11,13,25,26,27,28]. In the present study, we explore the reported phenomenon of the absence of the inner perianth whorl (corolla) in female flowers of some species of *Eriocaulon*, e.g., [9,29]. Complete loss of only one perianth whorl is a rare condition in monocots, except some specialized lineages with pollination by wind or water. The loss of the inner perianth whorl has been studied in detail in *Trillium apetalon* (Melanthiaceae: Liliales), where the absence of the petal whorl results in altered positions of all subsequent floral whorls starting with the alternisepalous insertion of the outer whorl stamens [30]. A perianth composed of (2)3 tepals is known in a species of *Aspidistra* (Asparagaceae: Asparagales), but here it likely appeared by amalgamation of the two whorls and strongly decreased merism rather than a loss of the inner whorl [31]. Stützel and Gansser [32] documented trimerous and tricyclic flowers with complete loss of petals as well as outer whorl stamens in *Lachnocaulon*, a North American genus of Eriocaulaceae-Paepalanthoideae. After the loss of two adjacent whorls of the original pentacyclic flower, normal alternation of whorls is maintained in such flowers of *Lachnocaulon*.

Apart from *Lachnocaulon*, the absence of petals has been reported for female flowers of the widespread tropical species *Eriocaulon cinereum* R.Br. (=*E. sieboldianum* Siebold and Zucc. ex Steudel) and taxonomically close species such as *E. redactum* Ruhland [9,29,33,34,35,36]. Female flowers of these plants possess small, reportedly free linear sepals whose number is variously counted as 0–3 [33,37], 1–2 [29], 2 [35,38,39], 2–3 [34,36] or 3 [9]. Their male flowers strongly differ from female ones in conspicuous sepals united almost up to their tips into a spathe-like structure and in the presence of petals. Inferring organ homologies in flowers with reduced or lost parts is not a trivial task. For example, Oliveira and Bove [40] described female flowers of *E. cinereum* as having linear petals possibly assuming a loss of sepals rather than petals in these plants. Steinberg [38] and Ghazanfar [39] illustrated two narrow structures below sepals in male flowers resembling what is described as sepals in female flowers.

One of the aims of the present study is to investigate whether the pattern of corolla reduction is the same in *Lachnocaulon* and the *Eriocaulon cinereum* group and whether the petals are indeed reduced in the latter. We use scanning electron microscopy (SEM) to infer organ number and position in young male and female flowers to understand developmental bases of the strong dimorphism of the two floral types. As soon as the first floral whorl is obviously crucial in establishing positions of subsequent whorls, the reported variation in calyx structure of female flowers may have an impact on position of all other organs, including the gynoecium. Trimerous gynoecia of *Eriocaulon* normally differ in orientation relative to the flower subtending bract from those in typical monocot flowers (Figure 1A,B). In the present study, we aimed to reveal whether this unusual gynoecium orientation is present in the *Eriocaulon cinereum* group and how stable is the pattern of gynoecium orientation here. Our results allow a broader discussion of some aspects of flower development in Eriocaulaceae. Earlier detailed SEM-based studies of flower development in *Eriocaulon* were performed on a species having regular trimerous flowers with well-developed perianth in both sexual types [13,23,41]. Obviously, more taxa should be studied in a genus with such an enormous variation of definitive flower morphology. The present study is partially filling this gap.

The plants studied here fit the concept of *E. redactum* (a member of the *E. cinereum* group) adopted in the Indian literature. *Eriocaulon redactum* is considered to be endemic to western parts of Peninsular India [29]. Cook [42] adopted a broad taxonomic concept and placed *E. redactum* in synonymy of *E. cinereum* s.l., but subsequent authors highlighted a need of recognizing it as closely related, but distinct species [29,35,43]. A wide species concept of *E. cinereum* is recently questioned by Larridon et al. [44] based on a global molecular phylogenetic study of *Eriocaulon*. Obviously, a taxonomic revision of the entire *E. cinereum* complex is needed, but it is out of the scope of the present study, which is aimed in resolving issues of flower structure and development. The choice of material for our study is appropriate, because *E. redactum* is distinguished in the taxonomic literature [9,29,35] by a greater degree of perianth reduction in female flowers (hence the name). A study of the most reduced form allows inferring the limits of perianth reduction in the group.

To provide a robust source of comparative data, we also investigated developmental morphology in two species of *Eriocaulon* with three sepals and three clearly visible petals, *E. dalzellii* Koernicke and *E. xeranthemum* Martius. Based on morphology, *E. dalzellii* has been classified in the same section as *E. cinereum* and *E. redcatum*; these species share the occurrence of white or pale yellow anthers [29]. *Eriocaulon xeranthemum* has black anthers and has been classified in a different section [29].

## 2. Results

### 2.1. Eriocaulon redactum

**Figure 2:**
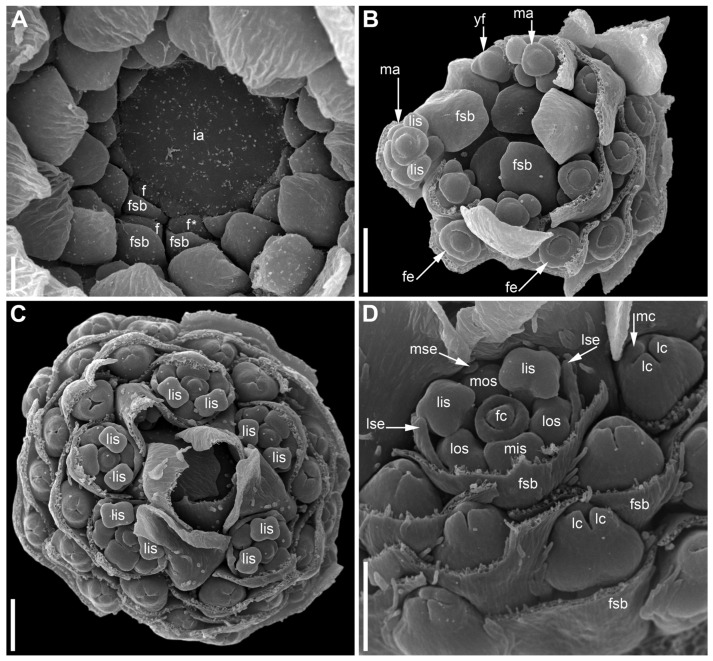
*Eriocaulon redactum*. Inflorescence structure (SEM). (**A**) Top view of distal part of very young inflorescence. (**B**,**C**) Top views of inflorescences at successive developmental stages. Subtending bracts of outer flowers are partially removed. Differences between functionally male and functionally female flowers are less pronounced in (**B**) than in (**C**) because of the earlier developmental stage. (**D**) Detail of (**C**) with a male and several female flowers. f, flower primordium or very young flower; fc, flower center; fe, functionally female flower; fsb, flower-subtending bract; ia, inflorescence apex; lc, lateral carpels; lis, lateral inner whorl stamens; los, lateral outer whorl stamens; lse, lateral sepals; ma, functionally male flower; mc, median carpel; mis, median inner whorl stamen; mos, median outer whorl stamen; mse, median sepal; yf, young flower (sexual status cannot be recognized yet). Scale bars = 30 μm (**A**), 100 μm (**B**–**D**).

**Figure 3:**
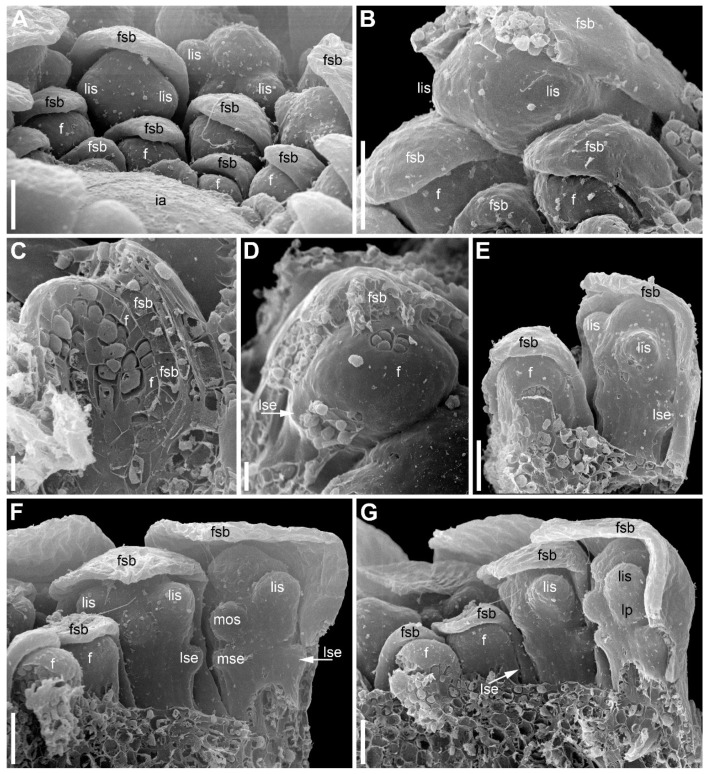
*Eriocaulon redactum*. Early flower development (SEM). These flowers are most likely female (based on their position in inflorescence). (**A**) Inflorescence apex with flowers at different developmental stages. (**B**) Three young flowers. (**C**) Flower before initiation of floral organs. The flower and its subtending bract are cut vertically to show their closely appressed adjacent surfaces. (**D**) Calyx initiation. (**E**) Two flowers at different developmental stages. The left flower is apparently before organ initiation. (**F,G**) Two views of a dissected inflorescence with four flowers at successive developmental stages. f, flower primordium or very young flower; fsb, flower-subtending bract; ia, inflorescence apex; lis, lateral inner whorl stamens; lp, lateral petals; lse, lateral sepals; mse, median sepal; mos, median outer whorl stamen. Scale bars = 30 μm (**A**,**B**,**E**–**G**), 10 μm (**C**,**D**).

The stalked inflorescence (Figure 2) possesses a short and broad receptacle covered by spirally arranged involucral bracts followed by numerous flower-subtending bracts. The flower-subtending bracts are spatulate (Figure 2B). They elongate early in development to cover the developing flowers (Figure 2A–C and Figure 3). In our material, most flowers in the inflorescence (including all peripheral ones) are functionally female and only about 5–6 flowers of several dozen are functionally male. Functionally male flowers form a series at the mid-level of the inflorescence (Figure 2C). When speaking of functionally male and functionally female flowers, we mean the presence of sterile organs of the opposite sex. The two flower types are called below for brevity as ‘male’ and ‘female’.

The inflorescence apex is flat and wide, about 6.5 times wider than bract primordia (Figure 2A). The floral primordia appear soon after initiation of flower-subtending bracts and before the next younger bract appears in the sector of the flower-subtending bract (Figure 2A and Figure 3A). The flower primordia are elliptic, strongly broadened in the transversal plane (f in Figure 2A). The young flowers soon become triangular in outline with an obtuse angle towards the inflorescence apex, but no floral organs can be traced at this stage (f* in Figure 2A). Then the young flowers exhibit a strong vertical growth and become nearly cylindrical (Figure 3E). Flower growth is coordinated with growth of the flower-subtending bract, and the bract remains tightly appressed to the flower (Figure 3C,E). The sepals are very small at initiation while the remaining floral apex is massive and flat (Figure 3D,G).

**Figure 4:**
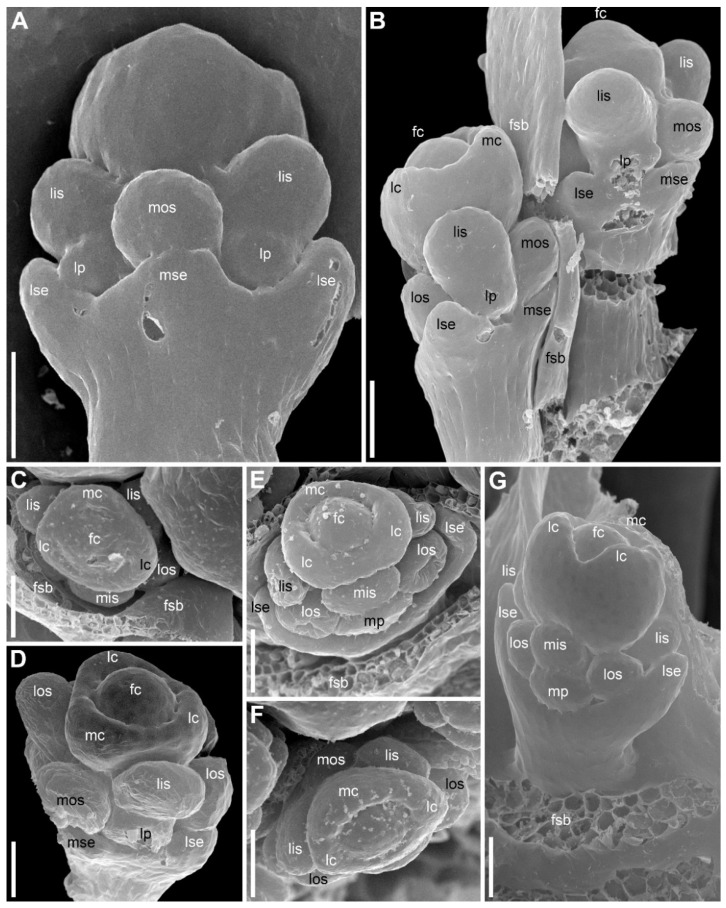
*Eriocaulon redactum*. Young functionally female flowers (SEM). (**A**) Flower viewed from the adaxial side. Massive distal part of the flower above stamens is the gynoecium. The flower is at the same stage of development as in the left flower in (**B**), but the floral center cannot be seen in this view. (**B**) Fragment of an inflorescence cut radially. The center of the inflorescence is at the right side. Two flowers are visible. The left flower is older than the right flower. Subtending bract of the left flower is removed. (**C**) Top view of flower at a stage intermediate between those of the two flowers in (**B**). (**D**–**G**) Different views of flowers at slightly younger (**F**) or about the same (**D**,**E**,**G**) stage as the older flower in (**B**). (**D**) Oblique side view. (**E**) Top-abaxial view. (**F**) Top-adaxial view. (**G**) Abaxial view. fc, floral center; fsb, flower-subtending bract; lc, lateral carpels; lis, lateral inner whorl stamens; los, lateral outer whorl stamens; lp, lateral petals; lse, lateral sepals; mc, median carpel; mis, median inner whorl stamen; mos, median outer whorl stamen; mp, median petal; mse, median sepal. Scale bars = 30 μm.

**Figure 5:**
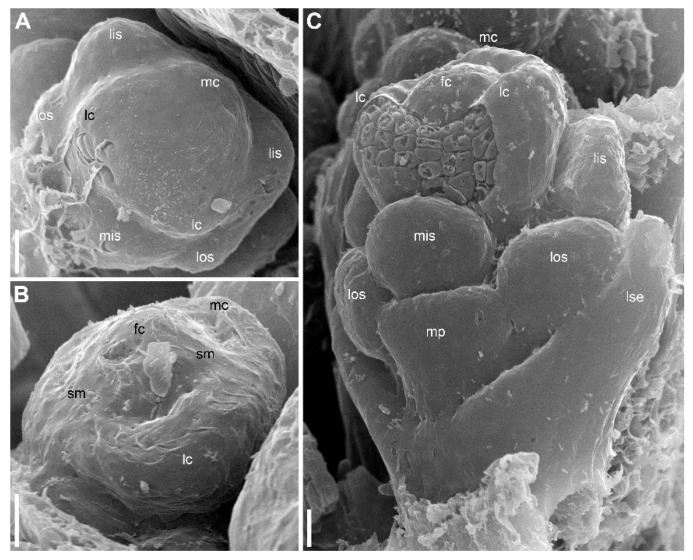
*Eriocaulon redactum*. Early gynoecium development (SEM). (**A**) The earliest evidence of gynoecium development. (**B**) Young gynoecium with shallow carpel locules. (**C**) Flower with dissected gynoecium. fc, floral center; lc, lateral carpels; lis, lateral inner whorl stamens; los, lateral outer whorl stamens; lse, lateral sepal; mc, median carpel; mis, median inner whorl stamen; mp, median petal. Scale bars = 10 μm.

At the earliest stages when male and female flowers can be distinguished from each other, both receptacle and pedicel are considerably elongated (Figure 4A). Observations of young female flowers (Figure 4 and Figure 5) clearly show their regular trimerous pentacyclic nature as outlined in Figure 1C. Note that organ abbreviations introduced in Figure 1C are then used consistently in all subsequent figures.

**Figure 6:**
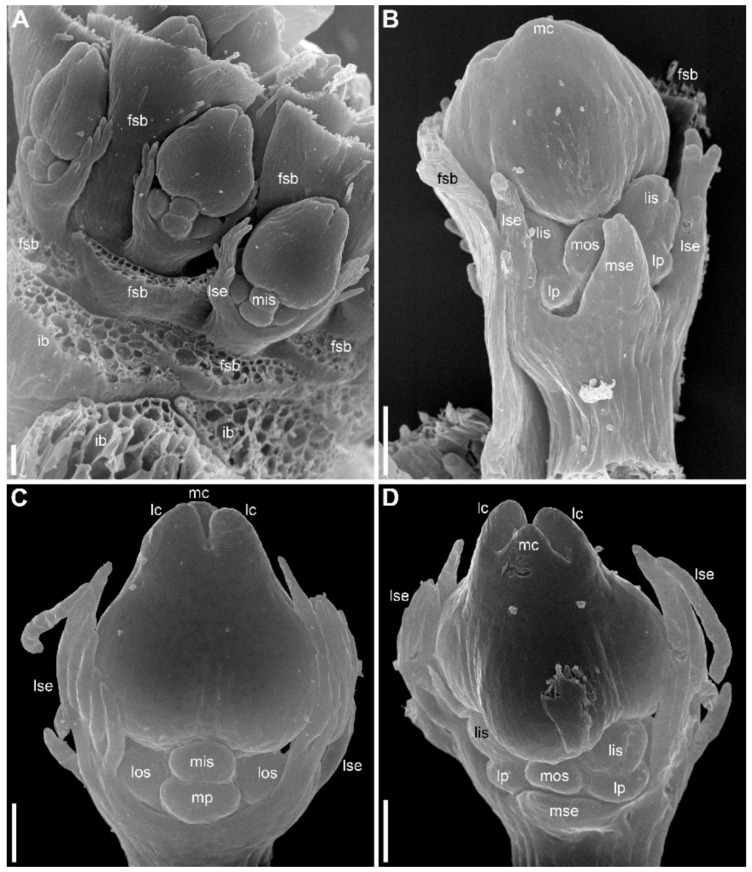
*Eriocaulon redactum*. Functionally female flowers at mid developmental stages. (**A**) Detail of inflorescence with involucral bracts and flower-subtending bracts removed to show three flowers. (**B**) Adaxial view of flower with distal part of flower-subtending bract removed. (**C**,**D**) Abaxial (**C**) and adaxial (**D**) views of another flower. The flower in (**C**,**D**) is on a slightly later stage than the flower in (**B**) based on hair development on lateral sepals, but the median sepal is shorter than in (**B**), apparently illustrating developmental plasticity of the median sepal. fsb, flower-subtending bract; ib, involucral bract; lc, lateral carpels; lis, lateral inner whorl stamens; los, lateral outer whorl stamens; lp, lateral petals; lse, lateral sepals; mc, median carpel; mis, median inner whorl stamen; mos, median outer whorl stamen; mp, median petal; mse, median sepal. Scale bars = 30 μm.

**Figure 7:**
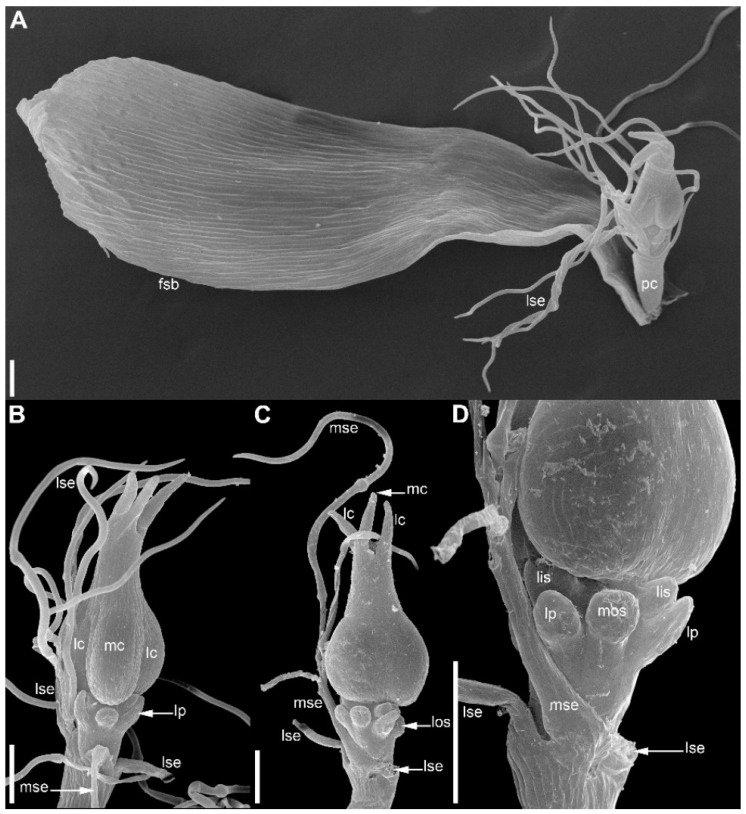
*Eriocaulon redactum*. Preanthetic functionally female flowers (SEM). (**A**) Side view of flower with its subtending bract. (**B**) Flower in adaxial view. (**C**) Oblique adaxial view of flower with well-developed median sepal (one of the lateral sepals removed, another partially removed). (**D**) Detail of (**C**). fsb, flower-subtending bract; lc, lateral carpels; lis, lateral inner whorl stamens; los, lateral outer whorl stamens; lp, lateral petals; lse, lateral sepals; mc, median carpel; mos, median outer whorl stamen; mse, median sepal; pc, flower pedicel. Scale bars = 100 μm.

**Figure 8:**
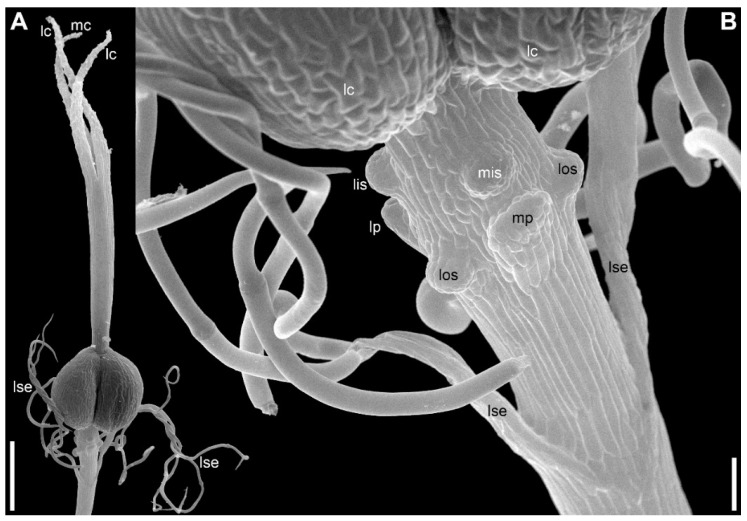
*Eriocaulon redactum*. Anthetic functionally female flower, adaxial view (SEM). (**A**) General view. (**B**) Detail of elongate receptacle with sepals, petals, staminodia and basal portion of gynoecium. lc, lateral carpels; lis, lateral inner whorl stamens; los, lateral outer whorl stamens; lp, lateral petals; lse, lateral sepals; mc, median carpel; mis, median inner whorl stamen; mp, median petal. Scale bars = 300 μm (**A**), 30 μm (**B**).

**Figure 9:**
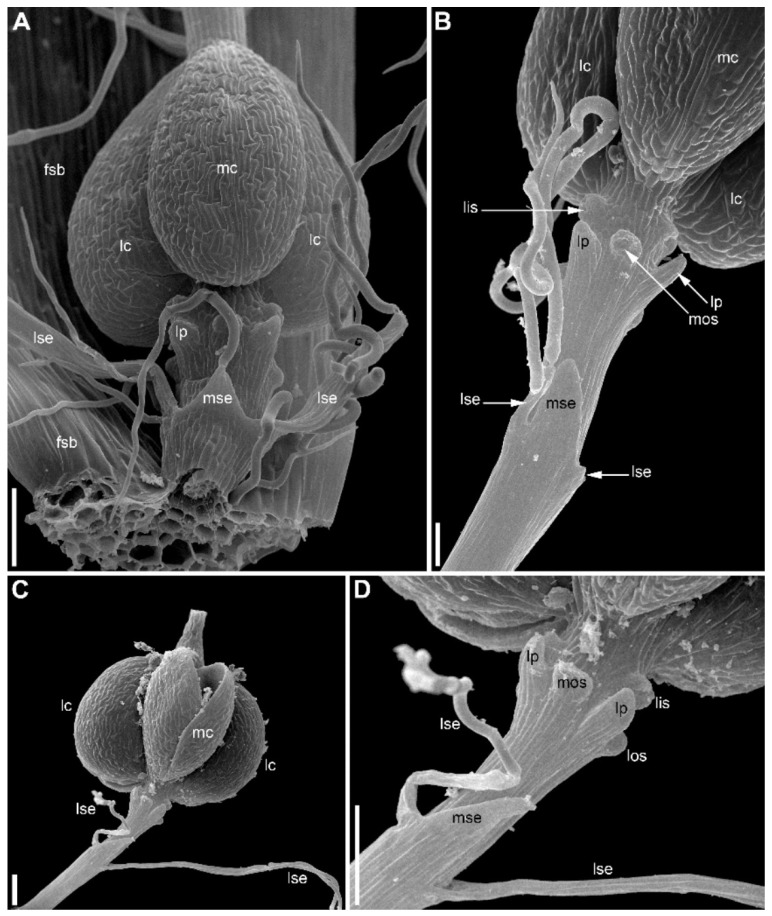
*Eriocaulon redactum*. Anthetic (**A**) and postanthetic (**B**) functionally female flowers and fruit (**C**,**D**), SEM. (**A**) Flower (except style and stigma), adaxial view. Median sepal triangular, with a hair at the top. Lateral sepals ribbon-like, each with several hairs. (**B**) Adaxial view, basal portion of flower. One lateral sepal removed. Median sepal elongate-triangular, glabrous. (**C**) Entire fruit, adaxial view. (**D**) Detail of (**C**) showing perianth and staminodia. fsb, flower-subtending bract; lc, lateral carpels; lis, lateral inner whorl stamens; los, lateral outer whorl stamens; lp, lateral petals; lse, lateral sepals; mc, median carpel; mos, median outer whorl stamen; mse, median sepal. Scale bars = 100 μm.

The calyx of female flowers always includes three sepals, two in lateral positions and the third in a median adaxial position (Figure 4A,B and Figure 5C). These are equal in size in very young flowers (Figure 4A,B). The angle between the two lateral sepals is close to 180° (Figure 4A,E,G). Even young sepals are basally united to form a complete calyx tube (Figure 4A,E). The young sepals are short and triangular and do not enclose the inner floral organs. The aestivation is apert. With subsequent development, the lateral sepals synchronously elongate so that their body almost reaches the level of the base of the gynoecium, and long hairs appear along the sepal margin in its distal part (Figure 6). The hairs are uniseriate and multicellular (Figure 6C,D and Figure 7). The median sepal is delayed in development. The degree of its developmental retardation varies, which can be illustrated by the following example. The lateral sepals in Figure 6B are at a younger stage than those in Figure 6D (as evidenced by relative lateral sepal/gynoecium length and degree of hair development), but the median sepal is shorter and less differentiated in Figure 6D than in the Figure 6B. Note that the flower in Figure 6B also shows a younger stage of gynoecium development than that in the Figure 6C,D. In preanthetic flowers (Figure 7), the lateral sepal body is linear, but short, not exceeding the gynoecium base, but the sepal hairs elongate considerably and much exceed the carpel tips (Figure 7A,B). Structure of the median sepal varies in preanthetic flowers. For example, its body can be short and triangular with only one distal hair (Figure 7B) or linear and bearing a group of long distal hairs (Figure 7C,D). Sepals of anthetic flowers (Figure 8) remain the same as before (Figure 7), but with the extensive elongation of the style and stigmas the sepal hairs appear much shorter than the gynoecium (Figure 8A). Definitive morphology of the median sepal also varies. For example, it is short, triangular and bears a long distal hair in Figure 9A but elongate-triangular and glabrous in Figure 9B. The calyx tube is usually conspicuous albeit short (Figure 9A). Sometimes, due to uneven elongation of the flower axis, the sepals are inserted at slightly different levels (Figure 9D).

There are three petals in female flowers (Figure 4D,E,G, Figure 6B–D, Figure 7B,D and Figure 8B), one of which is median abaxial and two are transverse-adaxial (here called lateral petals). Definitive petals are small (c. 40 μm wide and 100 μm long), glabrous, cylindrical or somewhat flattened structures (Figure 8B and Figure 9B). There are six sterile stamens (staminodes) arranged in two whorls. The outer whorl has a median adaxial stamen that lies in the radius of the median sepal (Figure 4A,B,F, Figure 6D, Figure 7B,D and Figure 9B,D) and two transverse-abaxial stamens (here called lateral outer stamens), that do not lie in the radii of the lateral sepals, but are closer to the abaxial side of the flower (Figure 1C, Figure 4B–G and Figure 6C). The outer whorl stamens are not united with other organs throughout development. The inner whorl stamens appear in the petal radii. Each petal and a stamen in its radius develop from a bilobed common primordium, where the petal lobe is much smaller than the stamen lobe (Figure 3G and Figure 4A,B). The petal part is retarded in development and thus not conspicuous at the following stages (Figure 3B,E).

The gynoecium is syncarpous with three carpels. The median carpel is adaxial (Figure 4B–G). The first evidence of carpel initiation can be seen when the floral apex becomes triangular in outline (Figure 5A). When the three carpels are clearly recognizable (right hand flower in Figure 4B), a massive hemispherical floral center remains conspicuous. The morphological nature of the floral center is considered in Discussion. Soon, a triangular rim embracing the floral center appears (Figure 4C–F and Figure 5B,C). The massive angles of the rim are dorsal parts of the three carpels. With extensive intercalary growth of the rim and underlying tissue, a massive trilocular ovary with a pendent ovule in each locule and a style are formed. There is a transient stage when the trilocular nature of the gynoecium with yet shallow locules divided by incipient septa can be seen without a dissection (Figure 5B). The dorsal parts of the carpels grow to join above the floral center (Figure 6) and finally develop stigmas. In preanthetic flowers (Figure 7), the trilobed ovary is slightly longer than the style and the stigmatic branches are as long as the style. The style and stigmas much elongate up to anthesis (Figure 8A). In fruit, each ovary locule dehisces dorsally to release a seed (Figure 9C).

The pedicel and the flower receptacle continue their elongation during flower development. In early stages, all floral organs are closely arranged and appressed to each other (Figure 4 and Figure 6). In preanthetic flowers, an internode between the calyx and the corolla (called anthophore) is conspicuous (Figure 7B–D). The anthophore further elongates by anthesis (Figure 8B), along with elongation of an internode between the androecium and gynoecium (called gynophore). Also, a short internode is conspicuous between the levels of the petals and in the inner whorl stamens (Figure 8B and Figure 9B,D). The elongation of the receptacle is more extensive on the adaxial side. As a result, the locule of the median adaxial carpel is inserted slightly higher up than two other locules in anthetic flowers (Figure 9A,B). Furthermore, the median adaxial outer whorl stamen is inserted slightly above the petals (Figure 7C,D and Figure 9A,B), whereas the lateral outer whorl stamens remain at the petal level (Figure 7C and Figure 8B).

**Figure 10:**
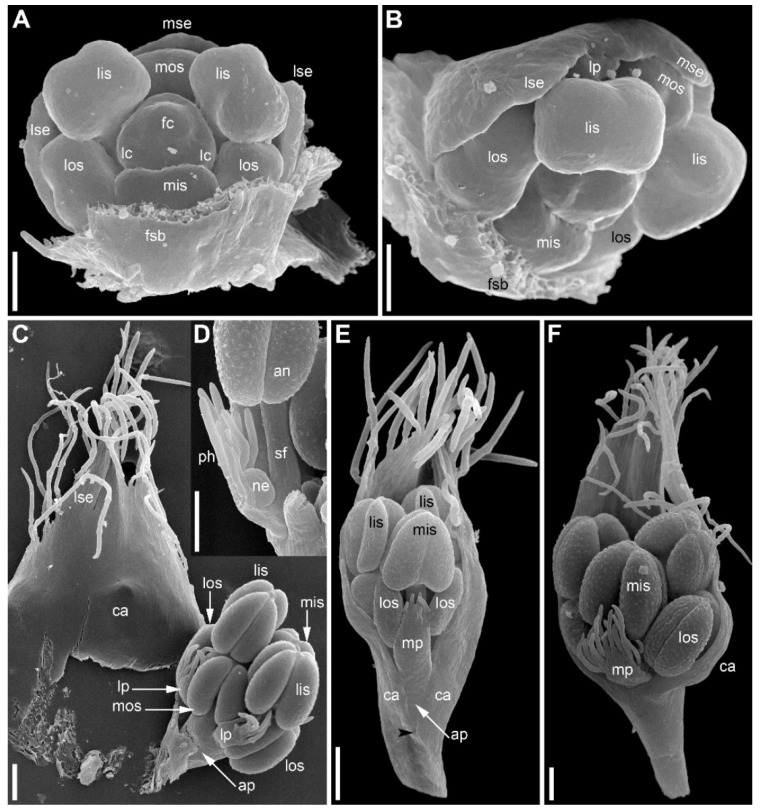
*Eriocaulon redactum*. Functionally male flowers (SEM). (**A**,**B**) Young flower. (**A**) Top view. (**B**) Oblique side view. (**C**) Preanthetic flower with calyx removed and mounted nearby. (**D**) Detail of dissected flower with side view of a petal and an inner whorl stamen in its radius. (**E**,**F**) Preanthetic flowers seen from the abiaxial side, (**E**) is younger and smaller than (**F**) as can be inferred from scale bars. Calyx tube is split up to the level indicated by arrowhead in (**E**), so that a portion of the anthophore is visible. Calyx tube in (**F**) encloses the anthophore completely. These differences are related to developmental plasticity of calyx. an, anther; ap, anthophore; fc, floral center; fsb, flower-subtending bract; lc, lateral carpels; lis, lateral inner whorl stamens; los, lateral outer whorl stamens; lp, lateral petals; lse, lateral sepals; mis, median inner whorl stamen; mos, median outer whorl stamen; mp, median petal; mse, median sepal; ne, nectary; ph, petal hairs; sf, stamen filament. Scale bars = 30 μm (**A**,**B**), 100 μm (**C**–**F**).

Organ number and arrangement in male flowers (Figure 10) are the same as in female flowers and follow the diagram in Figure 1C. Like in female flowers, the outer whorl lateral stamens do not lie in the radii of the lateral sepals (Figure 10A,B), because the angle between the lateral sepals is about 180º whereas the angle between the outer lateral stamens is about 120°. The sepals are basally united to form a calyx tube, which is much longer on the adaxial side forming a distally trilobed spathe-like structure. The lobes develop multicellular, uniseriate marginal hairs (Figure 10C,E,F). The degree of tube development on the abaxial side varies (Figure 10E,F), but at least in some flowers the tube extends up to the median petal base thus completely enclosing the anthophore (Figure 10F). The petals are shorter than the stamens. Their body is triangular in outline, bearing numerous long multicellular, uniseriate hairs. The inner whorl stamens are longer than the outer whorl stamens (Figure 10C,E,F). Furthermore, on earlier stages, the lateral inner whorl stamens appear to exceed the median inner whorl stamen (Figure 2B–D and Figure 10A,C). There is a sterile gynoecium whose early development is similar to that of the fertile gynoecium of female flowers (Figure 2B,C and Figure 10A,B). A stage when incipient septa between the ovary locules are visible without a dissection is observed in male flowers (Figure 2D). Male and female flowers are very similar to each other early in development, but after the appearance of a complete gynoecium rim embracing the floral center the two types of flowers can be distinguished by relative stamen and gynoecium size. Compare two flowers labelled as male and two flowers labelled as female in Figure 2B. The female flowers show more developed gynoecia (more pronounced ovary wall around the floral center), but much smaller stamens than in the male flowers.

### 2.2. Species with Petals Conspicuous in Anthetic Female Flowers

**Figure 11:**
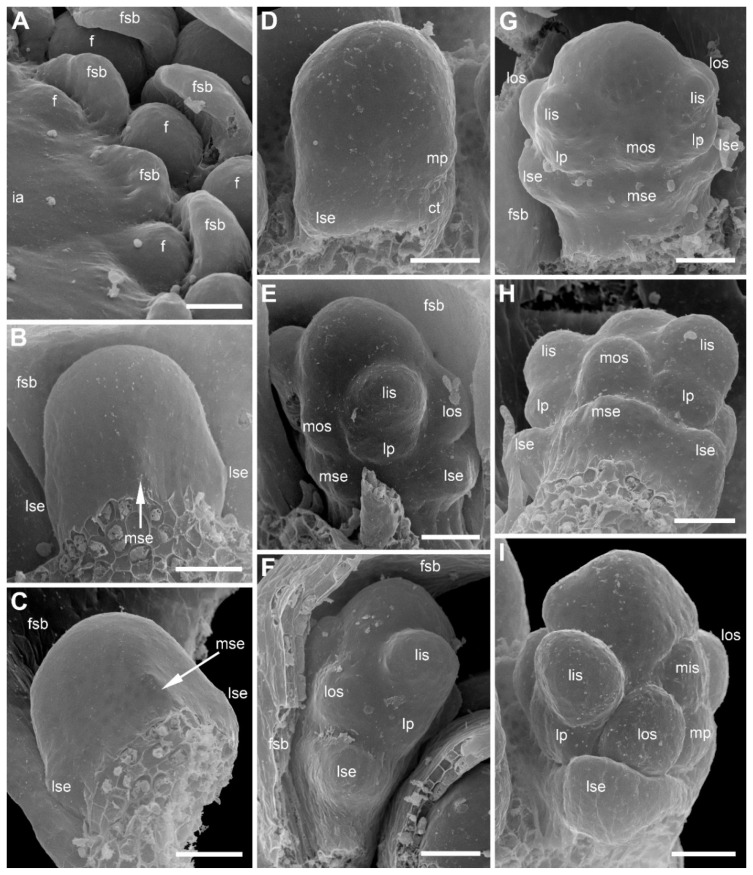
*Eriocaulon dalzellii*. Early flower development (SEM). Functionally male and functionally female flowers cannot be distinguished at these stages. (**A**) Detail of inflorescence apex with just initiated flower-subtending bracts and flowers. (**B**) Adaxial view of flower with sepals just initiated. The floral apex is conspicuously dome-shaped and extremely large compared to the tiny sepal primordia. (**C**) Stage similar to that in (**B**), the flower is removed from the inflorescence axis and seen in an adaxial-bottom view. (**D**) Flower with first evidence of corolla development, abaxial view. A narrow calyx tube is formed. (**E**–**H**) Flowers with all organs except carpels initiated. (**E**) Obliquely adaxial view. (**F**) Lateral view. (**G**) Adaxial view. (**H**) Adaxial-bottom view. (**I**) Flower with floral apex triangular in outline, which is the first manifestation of carpel development. f, flower primordium or very young flower; ct, calyx tube; fsb, flower-subtending bract; ia, inflorescence apex; lis, lateral inner whorl stamens; los, lateral outer whorl stamens; lp, lateral petals; lse, lateral sepals; mis, median inner whorl stamen; mos, median outer whorl stamen; mp, median petal; mse, median sepal. Scale bars = 30 μm.

**Figure 12:**
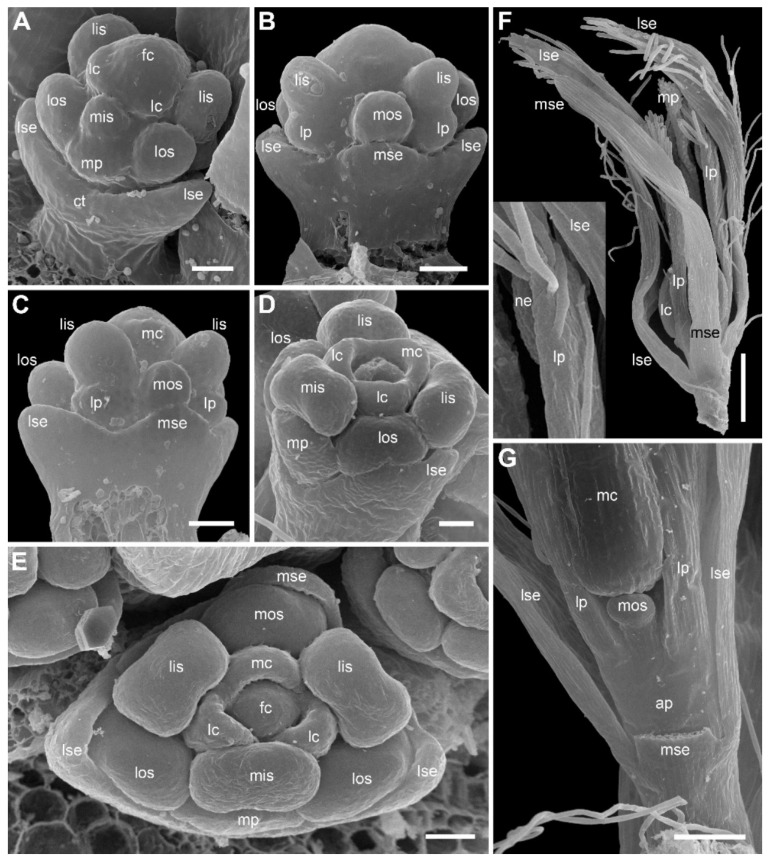
*Eriocaulon dalzellii.* Flowers at various stages of carpel and stamen development (SEM). (**A**–**C**) Stage when functionally male and functionally female flowers cannot yet be distinguished. (**A**) Abaxial view. (**B**) Adaxial view. (**C**) Obliquely adaxial view. (**D**,**E**) Functionally male flowers. (**D**) Obliquely abaxial view. (**E**) Top view, abaxial side bottom. (**F**,**G**) Fully formed preanthetic female flowers. (**F**) Adaxial view. Inset, magnified detail of right hand lateral petal showing its nectary. (**G**) Detail of adaxial view, median sepal removed to show one of six staminodia. ap, anthophore; fc, floral center; lc, lateral carpels; lis, lateral inner whorl stamens; los, lateral outer whorl stamens; lp, lateral petals; lse, lateral sepals; mc, median carpel; mis, median inner whorl stamen; mos, median outer whorl stamen; mp, median petal; mse, median sepal; ne, nectary. Scale bars = 30 μm (**A**–**E**), 100 μm (**G**), 300 μm (**F**).

**Figure 13:**
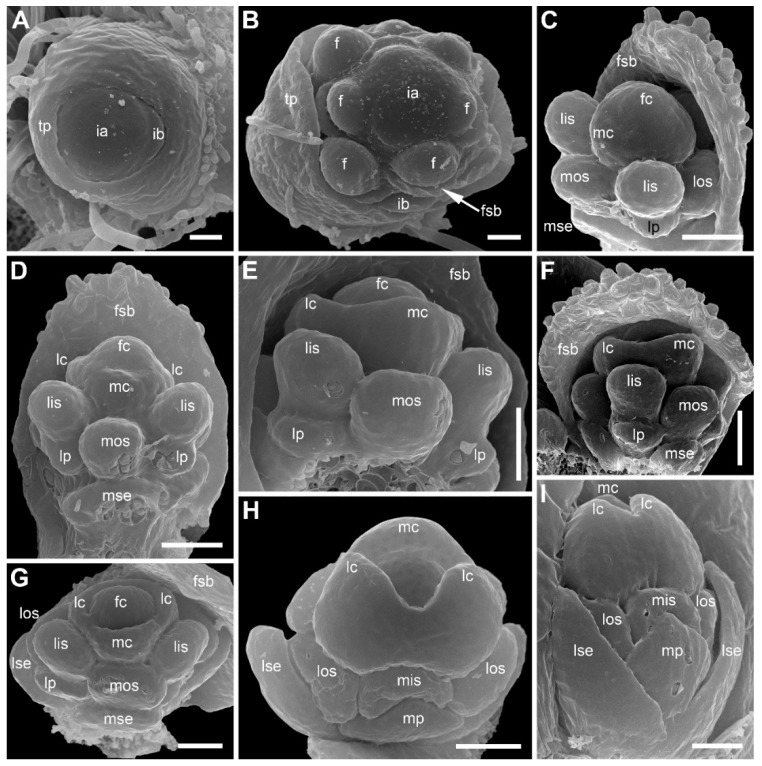
*Eriocaulon xeranthemum*. Inflorescence and flower development (SEM). (**A**) Inflorescence initiation. The inflorescence apex is surrounded by a tubular phyllome (a common feature of *Eriocaulon* species). The first involucral phyllome is initiated. (**B**) Flower initiation. The inflorescence is few-flowered compared to those of *E. dalzellii* and *E. redactum*. (**C**, **D**) Flowers with the earliest stage of carpel development; functionally male and functionally female flowers cannot yet be distinguished. (**C**) Lateral view. (**D**) Adaxial view. (**E**–**I**) Functionally female flowers. (**E**–**G**) Flowers with tips of the carpels not yet exceeding the floral center. (**E**) Adaxial view; median sepal removed. (**F**) Oblique adaxial view. (**G**) Adaxial-top view. (**H**, **I**) Flowers with tips of the carpels overtopping the floral center. (**H**) Abaxial-top view. (**I**) Oblique abaxial view. f, flower primordium or very young flower; fc, floral center; fsb, flower-subtending bract; ia, inflorescence apex; ib, involucral bract; lc, lateral carpels; lis, lateral inner whorl stamens; los, lateral outer whorl stamens; lp, lateral petals; lse, lateral sepals; mc, median carpel; mis, median inner whorl stamen; mos, median outer whorl stamen; mp, median petal; mse, median sepal; tp, tubular phyllome. Scale bars = 30 μm.

Flower development of *E. dalzellii* (Figure 11 and Figure 12A–E) and *E. xeranthemum* (Figure 13) is similar to that in *E. redactum*. Functionally male and functionally female flowers cannot be distinguished from each other before formation of a complete gynoecium rim embracing the floral center (Figure 11, Figure 12A–C and Figure 13B–D). Young flowers of the two species show no differences from each other and from those of *E. redactum*.

**Figure 14:**
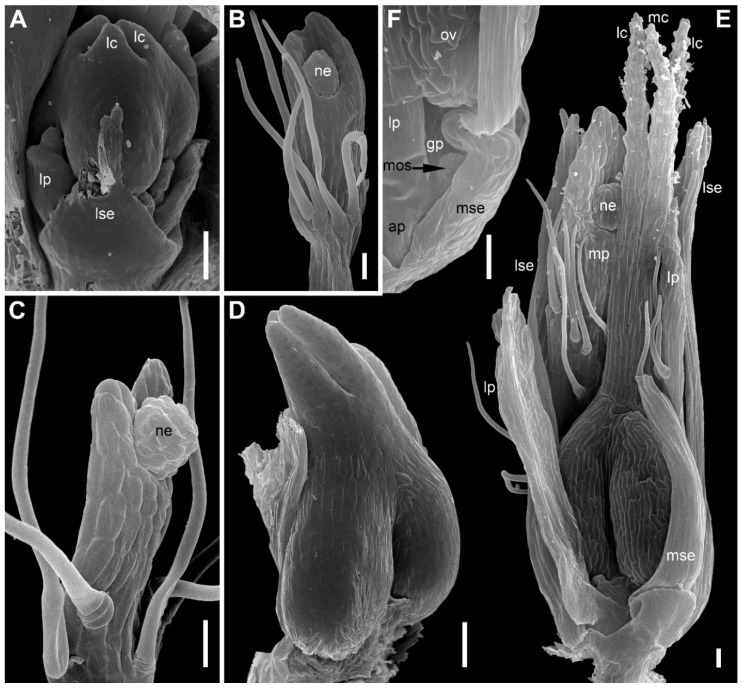
Functionally female flowers of *Eriocaulon xeranthemum* (SEM). (**A**) Lateral view of flower at the stage of petal nectary differentiation. The median petal is partly removed, but a lateral petal is well visible. Its apex will form a nectary. There is a belt below the incipient nectary that will form a secondary margin of the petal. (**B**) Petal with almost entire secondary margin above the nectary; adaxial view. (**C**) Petal with bifid secondary margin; oblique adaxial view. (**D**) Gynoecium with 3-lobed ovary, still short style and three stigmas yet lacking papillae. (**E**) Anthetic flower; oblique adaxial view. (**F**) Detail of another anthetic flower showing proximally folded medial sepal and relative position of the fold and a staminode (median outer whorl stamen). ap, anthophore; gp, gynophore; lc, lateral carpel; lp, lateral petal; lse, lateral sepal; mc, median carpel; mos, median outer whorl stamen; mp, median petal; mse, median sepal; ne, nectary; ov, ovary. Scale bars = 30 μm.

**Figure 15:**
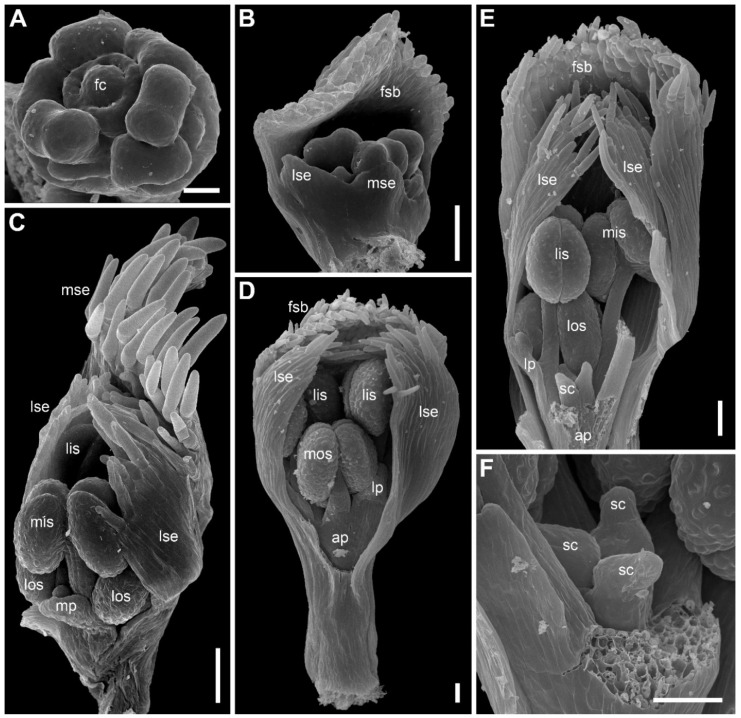
Functionally male flowers of *Eriocaulon xeranthemum* (SEM). (**A**) Young stage when stamens are longer than sepals. (**B**) Adaxial view of flower with flower-subtending bract. Calyx tube is visible. Sepal hairs are not yet initiated. (**C**–**E**) Preanthetic flowers. (**C**) Flower in oblique abaxial view. (**D**) Adaxial view; median sepal removed. (**E**) Adaxial view. The median sepal and some other organs are removed to show a sterile gynoecium, anthophore vertically dissected. A nectary of a lateral petal is visible. (**F**) Detail of dissected flower showing a sterile gynoecium. ap, anthophore; fc, floral center; fsb, flower-subtending bract; lis, lateral inner whorl stamens; los, lateral outer whorl stamens; lp, lateral petals; lse, lateral sepals; mis, median inner whorl stamen; mos, median outer whorl stamen; mp, median petal; mse, median sepal; sc, sterile carpel. Scale bars = 20 μm (**A**), 50 μm (**B**–**F**).

Later in development of female flowers, petal and sepal growth in length and width is much more extensive than in *E. redactum*. In the same way as in other species of the genus [41], a nectary develops from the apical part of a petal, but with formation of a secondary margin on the abaxial surface of the petal (Figure 14A), the nectary is being shifted onto the inner side of the petal (Figure 12F and Figure 14B,C,E). The secondary petal tip is usually entire, but sometimes strongly bilobed in female flowers of *E. xeranthemum* (Figure 14C). All six perianth members (three sepals and three petals) are conspicuous in mature flowers of *E. dalzellii* (Figure 12F) and *E. xeranthemum* (Figure 14E). The calyx tube remains extremely short, but recognizable in both species (Figure 12G and Figure 14E,F). The two species differ in patterns of receptacle elongation in female flowers. In *E. dalzellii* (Figure 12G) there is an anthophore (which is still shorter than in *E. redactum*), but no gynophore. A short gynophore and a short anthophore are both present in *E. xeranthemum* (Figure 14F). As a result, there is a gap between the bulging median carpel locule and a staminode in its radius in *E. xeranthemum*. The median sepal is often folded to fill this gap (Figure 14E,F). There is no gap between the median carpel locule and the staminode formed by the median outer whorl stamen in *E. dalzellii* (Figure 12G), and the median sepal is not folded in this species (Figure 12F). Young male flowers of *E. xeranthemum* (Figure 15A) and *E. dalzellii* (Figure 12D) are similar to each other and to those of *E. redactum*. Characteristic feature of *E. xeranthemum* is the absence of hairs on petals (Figure 15), which makes petal nectaries well-detectable (Figure 15C,E). The petal gland position is the same as in female flowers.

## 3. Discussion

**Stable groundplan shared by male and female flowers**. Contrary to earlier observations on *Eriocaulon redactum* based on light microscopy [9,29,35], our data based on SEM clearly demonstrate the presence of petals and staminodes in female flowers of this species. Moreover, we demonstrate that the organ number as well as organ positions are identical between male and female flowers. According to our data, both flower types possess five floral whorls and all whorls are trimerous. Earlier reports of the presence of less than three sepals are likely because of the very small size of all floral organs to describe them in detail using only light microscopy. This is the reason why the petals were previously regarded absent in female flowers. As soon as *E. redactum* has more reduced perianth of female flowers than *E. cinereum* s.str. (sepal body 0.05–0.2 mm in the former, 0.5–1.7 mm in the latter, [34,36,45], we believe that our conclusions might be plausible for the entire *E. cinereum* species group. Indeed, Kral [45] reported the occurrence of three spreading, peglike, minute appendages on the gynophore, and it is likely that these were petals. In the light of recent molecular phylogenetic data [44], the entire *E. cinereum* group needs a worldwide taxonomic revision. We highlight an urgent need of use of developmental data documented by scanning electron microscopy to assess variation of floral characters in the group.

Patterns of perianth reduction differ between *Lachnocaulon* [32] and the examined species of *Eriocaulon* (*E. redactum*). In the former, the corolla (as well as the outer whorl stamens) is not initiated at all in both male and female flowers. In the latter, all five floral whorls are initiated in flowers of both sexes, but further development of petals and stamens is suppressed in the female flowers. Literature data on the absence of petals in female flowers of some other *Eriocaulon* species such as *E. achiton* Koernicke, e.g., [9,29,34,35,36], require re-investigation using SEM and may also reflect the petal suppression rather than petal loss. The report of single whorled perianth for 14 Indian species of *Eriocaulon* [46] is based on an assumption of the ancestrally 3-whorled androecium in the genus with sterilization of the outer whorl stamens. This hypothesis does not fit the phylogenetic placement of Eriocaulaceae, as we do not know flowers with three tepals and nine stamens in three whorls in any other monocot family.

Our data fit well with the conclusion made previously for other Eriocaulaceae that the difference between male and female flowers appears relatively late in development [8,11,13,23]. The similarity of young male and female flowers in all three *Eiocaulon* species studied here is remarkable. All three petals and six stamens manifested in male flowers possess their counterparts in female flowers, though they are small and inconspicuous in female flowers of *E. redactum*. Early stages of gynoecium development are identical in male and female flowers. Differences in mature calyx structure between male and female flowers are especially strong in species studied here because the sepals of male flowers are united into a long adaxial spathe-like structure. Our data show, in contrast to earlier observations based on light microscopy, that the calyx tube is present in female as well as male flowers of all three examined species. The calyx tube of female flowers is short, but can be recognized since early developmental stages.

**Pedicel and receptacle elongation**. Flowers of *E. redactum* as well as some other species of *Eriocaulon* are distinctly pedicellate. Receptacle elongation is a common phenomenon in Eriocaulaceae. Typically, an elongated internode (anthophore [4]) is present between the calyx and corolla in Eriocaulaceae [11,13]. The anthophore is generally uncommon in angiosperms. Its presence is well-known in some members of the eudicot family Caryophyllaceae, e.g., [47] and recently documented in Geraniaceae [48,49,50]. Recognizing an anthophore is only possible in groups with stable perianth groundplan where sepal and petal homologies are clear. Indeed, the general stability of the two-whorled perianth among monocots allows precise identification of sepals and petals separated by anthophore in *Eriocaulon*. An internode between calyculus and outer tepals found in Tofieldiaceae (Alismatales) resembles an anthophore, but homologies of the calyculus are unclear [51].

Similar to other species of *Eriocaulon*, an anthophore is well developed in male flowers of the species studied here. The stalk between the calyx and the ovary in female flowers of *E. redactum* and other members of the *E. cinereum* complex was usually described as gynophore (a term describing a stalk between stamens and carpels). Since we documented the occurrence of petals and staminodes, we are able to conclude that both anthophore and gynophore are present in the species studied here. Moreover, we found a gap between the levels of insertion of petals and inner whorl stamens. Therefore, most internodes elongate in female flowers of *E. redactum*. An exception is the internode between the petals and the outer whorl stamens. The insertion of the median adaxial outer whorl stamen slightly above the adjacent petals is because of greater receptacle elongation on the adaxial side. Receptacle elongation is present in female flowers of *E. xeranthemum* and *E. dalzellii*, but it is less pronounced than in *E. redactum*. A short gynophore is present in *E. xeranthemum*, but not in *E. dalzellii*. The gynophore presence in *E. xeranthemum* creates a gap between the median adaxial staminode and the median carpel. This gap often contains a fold of the median sepal (Figure 14F). In our view, the fold appears because the upper portion of the median sepal is tightly appressed to the ovary. Sepal elongation is at least partly localized in its proximal part, and the sepal fills the available space between the ovary and the staminode. This is a good example of coordinated growth patterns of adjacent organs governed by physical constrains [52,53].

Elongation of more than one floral internode is an extremely rare condition among angiosperms. In the order Poales, such a condition is known in *Typha* (Typhaceae). We assume that it is plausible to consider bristles associated with gynoecium of *Typha* as phyllomes [54,55] rather than merely trichomes, e.g., [56,57]. As pointed out by Müller-Doblies [55], the bristles (‘perigone hairs’) of the female flower of *Typha* originate in 4 to 1 somewhat irregular whorls with internodes in between and a long stalk just below the carpel. The occurrence of up to four perianth whorls is intriguing, because this condition has no obvious parallels among other monocots. It is possible that the third and the fourth whorls of bristles, when present, are formed by staminodia rather than tepals and the flowers are thus pentacyclic as in most monocots.

A peculiar receptacle elongation is found in *Centrolepis* (Restionaceae s.l. or Centrolepidaceae, Poales). In *Centrolepis*, the receptacle elongation is strongly one-sided, which often allows spacing much more than the usual monocot number of three carpels. These form a single distorted whorl in *Centrolepis* [58,59]. The position of the median carpel at a slightly upper level than the lateral carpels in *E. redactum* resembles the condition found in *Centrolepis*, albeit in a less pronounced form.

Among Poales other than Eriocaulaceae, a gynophore occurs in flowers of Juncaceae and some Cyperaceae [60,61,62,63]. Elaborated gynophores forming a lobed cup below the gynoecium are known in Cyperaceae [60].

**Common petal–stamen primordia**. Common tepal(petal)–stamen primordia for pairs of organs inserted in the same radii are widespread among monocots, but their occurrence is homoplastic [2]. In Poales, common petal–stamen primordia are present in *Xyris* [11,20] and *Eriocaulon* ([8,11,23] and this study). The occurrence of common petal–stamen primordia in other genera of Eriocaulaceae, e.g., *Paepalanthus*, is controversial [11,28]. Remarkably, in most other monocots possessing common primordia, these occur only for inner whorl perianth members and corresponding stamens, even when the two perianth whorls are similar to each other in anthetic flowers (e.g.,*Veratrum*, Liliales, [2]; *Dioscorea*, Dioscoreales, [64]) or rarely for all perianth members and stamens on their radii. Formation of two whorls of common tepal–stamen primordia is documented in *Allium* (Asparagales: [65,66], sometimes takes place in *Tofieldia* (Alismatales), where this condition is unstable within a species [67] and possibly occurs in *Scheuchzeria*, Alismatales [2,68,69,70]. There is no convincing example in monocots where common primordia are formed for the outer perianth whorl and outer androecium whorl only, except when the inner whorl stamens are absent (Iridaceae: [71,72]).

Endress [2] suggested a link between the occurrence of common (inner) tepal–stamen primordia and delayed receptacle expansion in lilioid monocots. In monocots with delayed receptacle expansion and delayed carpel initiation, initiation of (inner) tepals and stamens takes place in a very rapid sequence, or almost simultaneously, leading to the appearance of common tepal–stamen primordia [3,73]. However, the data on *Eriocaulon* do not fit this theory, because the receptacle elongation is strong already at early developmental stages. The floral apex is remarkably long already at the stage of sepal initiation (e.g., Figure 11B). The occurrence of common primordia may be related to the fact that petal primordia are smaller than stamen primordia in *Eriocaulon* [23]. In Eriocaulaceae with separate petal and inner stamen primordia [11], petals are initiated simultaneously with stamens (*Leiothrix*) or even after the stamens (*Paepalanthus*). Petal initiation after the stamens is a rare example on non-acropetal flower development in angiosperms that is better known in a few eudicots [65,70,74,75]. According to Ronse De Craene and Smets [76], the appearance of common petal–stamen primordia found in various eudicot families could be interpreted in terms of a retardation in petal inception and slower growth of the petals, which is connected with strongly developed antepetalous stamen primordia. Apparently, Eriocaulaceae provide a similar example among monocots. The highly reduced tepals of Cyperaceae-Cyperoideae are initiated after stamen initiation [61]. Only outer whorl stamens are present in Cyperoideae. The question on potential occurrence of common tepal–stamen primordia in Cyperoideae should be further investigated. In our view, a common base of young tepal and stamen can be seen in a published image of *Eleocharis palustris* ([58], Figure 1K).

As pointed out by Endress [2] the occurrence of fusion between tepals (petals) and stamens in monocots is neither a necessary consequence of common primordia nor are common primordia an obligate precondition for fusion. Female flowers of *Eriocaulon redactum* provide an extreme example in this context. Indeed, even though common petal–stamen primordia are present, the petals and inner whorl stamens are not only free, but even divided by a well visible internode. In female flowers of some other species of the genus, there is a long stalk (androgynophore) between the corolla and the two whorls of staminodia [11,77].

**Sepal arrangement and flower orientation**. As outlined in the Introduction, flower orientation relative to the subtending bract in Eriocaulaceae is reverse to what is observed in other monocots lacking floral prophylls (Figure 1A,B). This difference is enigmatic and deserves special attention. Clearly, sepal initiation plays a key role in patterning of all other floral organs. A remarkable feature of lateral sepals documented here for all three investigated species of *Eriocaulon* is that their position is nearly transversal rather than transverse-abaxial as can be expected in a regular trimerous whorl (Figure 1C). As a result, the lateral outer whorl stamens (which are not displaced from the transverse-abaxial position) do not lie in the sepal radii. The same pattern is illustrated by Stützel [23] for another species of the genus. A comparison with *Xyris* is instructive. In both Xyridaceae and Eriocaulaceae, there is a tendency for a delayed initiation, reduction or evolutionary loss of the median sepal [11,17]. Still, the angle between the lateral (adaxial-transversal) sepals is about 120° in *Xyris*, just as expected in trimerous flowers. This pattern of lateral sepal arrangement in *Xyris* is clear even before median sepal initiation [11,20]. One may hypothesize that a change from adaxial-transversal to transversal position of lateral sepals in stem group of Xyridaceae resulted in a shift from abaxial to adaxial position of the median sepal along with associated alteration in positions of all other floral organs. On the other hand, the angle between the lateral tepals (and stamens) is about 180° in at least some Cyperaceae-Cyperoideae, but the entire flower orientation remains the same as in Xyridaceae and many other monocots [61].

Species of Eriocaulaceae and Xyridaceae with dimerous calyx and trimerous other floral whorls require detailed SEM-based developmental studies. The occurrence of the median sepal at least at the level of pre-pattering should be important for establishment of subsequent trimerous whorls. Our study of *E. redactum* is instructive in this respect. Despite the literature reports of flowers with dimerous calyx and trimerous gynoecium, all flowers studied here were isomerous.

Floral apex shape and time of flower initiation may be related to the pattern of sepal initiation found in *Eriocaulon*. It seems that flower primordia appear in axils of subtending bracts much earlier in *Eriocaulon* (present study) than in *Xyris* [20]. Flower primordia are recognizable in all three species studied here at stages before appearance of subsequent bracts in the sector of the flower-subtending bract (Figure 2A, Figure 11A and Figure 13B). The floral primordia are ellipsoid, strongly transversally elongate in top view. A highly remarkable feature of *Eriocaulon* and apparently other Eriocaulaceae is the strong vertical elongation of the floral apex before sepal initiation. The sepal primordia are much smaller than the remaining cylindrical floral apex. Among monocots, these features seem to be nearly restricted to Eriocaulaceae.

**Gynoecium: similarities with Restionaceae and Cyperaceae**. A feature of young gynoecia of *Eriocaulon* is the occurrence of a massive hemispherical floral center surrounded by a trilobed rim of the future outer wall of the gynoecium [23]. This feature is not recorded in two other ‘xyrid’ families of Poales, Xyridaceae [11,20] and Mayacaceae [78]. Morphological interpretation of the ‘floral center’ is problematic in *Eriocaulon* and other monocots possessing this structure as it could be viewed as congenitally united ventral parts of the three carpels, as remaining floral apex or as a combination of carpels and receptacle [3,79,80,81]. A massive convex floral center resembling that of *Eriocaulon* is found in another family of Poales with mostly unisexual (though normally dioecious) flowers, Restionaceae [81,82,83]. Restionaceae s.l. (including Anarthriaceae and Centrolepidaceae [84]) share with Eriocaulaceae the occurrence of a single pendent ovule per locule and dorsal (when any) fruit dehiscence [85,86,87]. The same is true for the members of the graminid clade with plurilocular gynoecia (Flagellariaceae, Joinvilleaceae, Ecdeiocoleaceae: [88,89]. These data may be interpreted as supporting placement of Eriocaulaceae as sister to restiids + graminids in the situation of still insufficient molecular evidence of relationships of the family [18,19].

The following difference in gynoecium development of Restionaceae and *Eriocaulon* can be traced (developmental data on Flagellariaceae, Joinvilleaceae and Ecdeiocoleaceae are incomplete). In Restionaceae, the trilocular nature of the ovary is clearly visible in top view of the gynoecium before its closure. In *Eriocaulon*, there is a transient stage with the locules visible without a dissection (Figure 5B), but then a continuous triangular rim of the outer wall of the gynoecium grows extensively around the massive floral center and hides the locules and septa. It is intriguing that some young stages of gynoecium development in *Eriocaulon* are extremely similar to those in Cyperaceae-Cyperoideae. In both cases, there is a central hemisherical bulge surrounded by incipient ovary wall [61,90]. Subsequent development is of course very different in Cyperaceae, because the central bulge develops into an ovule and the ovary remains unilocular [61,90].

## 4. Materials and Methods

Material of *E. redactum* was collected in a wild population in the campus of Shivaji University, Kolhapur, Maharashtra, India (voucher specimen: *S.R. Yadav,* 1 September 2019, MW). Samples of *E. dalzellii* and *E. xeranthemum* were collected near Abasaheb Marathe Arts and New Commerce, Science College, Ratnagiri, Maharashtra, India (voucher specimens: *A. Chandore*, 28 August 2019, MW). The material was fixed in 70% ethanol. For SEM, material was dissected in 70% ethanol and then transferred to 100% acetone using the following series: 96% ethanol (twice for 30 min), 96% ethanol: 100% acetone (1:1 *v*/*v*, 30 min), 100% acetone (three times 30 min). The material was critical point-dried using a Hitachi HCP-2 critical point dryer (Hitachi, Japan), then coated with gold and palladium using a Giko IB-3 ion-coater (Tokyo, Japan) and observed using a CamScan S-2 (Cambridge Instruments, London, UK) at the Laboratory of Electron Microscopy at the Biological Faculty of Moscow University.

## 5. Conclusions

Scanning electron microscopy should be used in taxonomic accounts of *Eriocaulon* to assess organ number and arrangement, especially for taxa with reduced perianth of female flowers such as *E. cinereum* and related species. The present study does not support earlier views on variation in sepal number in female flowers of *E. redactum.* The calyx is reduced, but sepal number and position are stable and fit the general pattern of Eriocaulaceae. The type of orientation of the trimerous flowers found in Eriocaulaceae can be related to the position of lateral sepals. Two types of corolla reduction are found in Eriocaulaceae: petal suppression (found in the present study) and complete loss of petals (*Lachnocaulon*: [32]). Patterns of receptacle elongation are similar in *Eriocaulon* and *Typha* (Typhaceae). Our comparison between the two groups showed a possibility of an updated morphological interpretation of the bristles surrounding female flowers in *Typha*, which requires further testing. In contrast to other monocots, formation of common petal–stamen primordia is not linked with delayed receptacle expansion, but apparently with slow early petal growth in *Eriocaulon*. Gynoecium development indicates similarities of Eriocaulaceae with restiids and graminids rather than with Xyridaceae. This observation is significant, because Eriocaulaceae and Xyridaceae are among relatively few monocot families with controversial placement in molecular phylogenetic studies [18,19].
